# Bruch’s membrane abnormalities in PRDM5-related brittle cornea syndrome

**DOI:** 10.1186/s13023-015-0360-4

**Published:** 2015-11-11

**Authors:** Louise F. Porter, Roberto Gallego-Pinazo, Catherine L. Keeling, Martyna Kamieniorz, Nicoletta Zoppi, Marina Colombi, Cecilia Giunta, Richard Bonshek, Forbes D. Manson, Graeme C. Black

**Affiliations:** Institute of Human Development, Centre for Genomic Medicine, Faculty of Medical and Human Sciences, University of Manchester, Manchester Academic Health Science Centre (MAHSC), Saint Mary’s Hospital, Oxford Road, Manchester, M13 9WL UK; Manchester Royal Eye Hospital, Central Manchester University Hospitals NHS Foundation Trust, Oxford Road, Manchester, M13 9WL UK; Department of Eye and Vision Science, Institute of Ageing and Chronic Disease, University of Liverpool, Liverpool, UK; Department of Ophthalmology, Unit of Macula, University and Polytechnic Hospital La Fe, Valencia, Spain; Histopathology, Central Manchester University Hospitals, NHS Foundation Trust, Manchester Royal Infirmary, Oxford Road, Manchester, M13 9WL UK; Division of Biology and Genetics, Department of Molecular and Translational Medicine, School of Medicine, University of Brescia, Brescia, Italy; Division of Metabolism, Connective Tissue Unit, University Children’s Hospital and Children’s Research Centre, (CRC) Zurich, Switzerland; Department of Histopathology, National Ophthalmic Pathology Service (NSOPS) Laboratory, Central Manchester University Hospitals, NHS Foundation Trust, Manchester Royal Infirmary, Oxford Road, Manchester, M13 9WL UK; Centre for Genomic Medicine, Central Manchester University Hospitals, NHS Foundation Trust, MAHSC, Saint Mary’s Hospital, Oxford Road, Manchester, M13 9WL UK

**Keywords:** Brittle cornea syndrome, ZNF469, PRDM5, Corneal rupture, Bruch’s membrane, Choroidal neovascularization

## Abstract

**Background:**

Brittle cornea syndrome (BCS) is a rare, generalized connective tissue disorder associated with extreme corneal thinning and a high risk of corneal rupture. Recessive mutations in transcription factors *ZNF469* and *PRDM5* cause BCS. Both transcription factors are suggested to act on a common pathway regulating extracellular matrix genes, particularly fibrillar collagens. We identified bilateral myopic choroidal neovascularization as the presenting feature of BCS in a 26-year-old-woman carrying a novel *PRDM5* mutation (p.Glu134*). We performed immunohistochemistry of anterior and posterior segment ocular tissues, as expression of PRDM5 in the eye has not been described, or the effects of PRDM5-associated disease on the retina, particularly the extracellular matrix composition of Bruch’s membrane.

**Methods:**

Immunohistochemistry using antibodies against PRDM5, collagens type I, III, and IV was performed on the eyes of two unaffected controls and two patients (both with Δ9-14 *PRDM5*). Expression of collagens, integrins, tenascin and fibronectin in skin fibroblasts of a BCS patient with a novel p.Glu134* *PRDM5* mutation was assessed using immunofluorescence.

**Results:**

PRDM5 is expressed in the corneal epithelium and retina. We observe reduced expression of major components of Bruch’s membrane in the eyes of two BCS patients with a *PRDM5* Δ9-14 mutation. Immunofluorescence performed on skin fibroblasts from a patient with p.Glu134* confirms the generalized nature of extracellular matrix abnormalities in BCS.

**Conclusions:**

PDRM5-related disease is known to affect the cornea, skin and joints. Here we demonstrate, to the best of our knowledge for the first time, that PRDM5 localizes not only in the human cornea, but is also widely expressed in the retina. Our findings suggest that ECM abnormalities in *PRDM5*-associated disease are more widespread than previously reported.

**Electronic supplementary material:**

The online version of this article (doi:10.1186/s13023-015-0360-4) contains supplementary material, which is available to authorized users.

## Background

Brittle cornea syndrome (BCS) is an autosomal recessive connective tissue disorder predominantly affecting the cornea, skin and joints [1–4]. Extreme corneal thinning (220–450 μm) (normal range 520–560 μm) is the hallmark of the condition and affected individuals are at high risk of corneal rupture, leading to irreversible blindness [1, 2]. Other ocular features include blue sclerae, keratoconus, keratoglobus and high myopia. Extra-ocular manifestations include deafness, joint hypermobility, skin hyperelasticity, arachnodactyly, and developmental dysplasia of the hip [2]. BCS has been described in patients in the absence of extra-ocular features [2] and diagnosis prior to ocular rupture is possible in the presence of a high index of clinical suspicion.

BCS results from mutations in one of two genes; *ZNF469,* encoding zinc finger protein 469 (BCS type 1 [MIM 229200]) [3] and *PRDM5,* encoding PR domain-containing protein 5 (BCS type 2 [MIM 614161]) [4]. Both ZNF469 and PRDM5 proteins are suggested to act on a common pathway regulating extracellular matrix (ECM) gene expression [2–5]. A previous study using chromatin immunoprecipitation (ChIP) - sequencing has shown a direct role for PRDM5 in the regulation of collagen genes [6].

A role for PRDM5 in bone development [6] and corneal development and maintenance [4] has been suggested. However, the exact localization of the protein in the human eye has not been described. We performed immunohistochemistry (IHC) in human eyes and found that PRDM5 localizes both to the corneal epithelium and the retina. Aiming to gain insights into a role for this protein in the retina, we examined the deposition of ECM proteins in the retinas of BCS patients with a *PRDM5* Δ9–14 mutation using IHC, and found ECM abnormalities within Bruch’s membrane. We also report abnormal expression of ECM components in fibroblasts from a BCS patient with high axial myopia and choroidal neovascularization carrying a novel p.Glu134* mutation in *PRDM5*, a patient who had not sustained a corneal rupture. These data suggest that ECM abnormalities in *PRDM5*-related disease are more widespread than previously reported, and suggest a role for PRDM5 in the retina.

## Methods

### Participants and clinical evaluation

Informed written consent was obtained and investigations conducted in accordance with the principles of the Declaration of Helsinki, with Local Ethics Committee approval (NHS Research Ethics Committee reference 06/Q1406/52). Patients with BCS P1 and P2, with *PRDM5* Δ9–14; and P3, with *PRDM5* p.Arg590* have been previously described [4]. Diagnosis of BCS in P4, with *PRDM5* p.Glu134*, was based on clinical examination and confirmed by mutation analysis of *ZNF469* and *PRDM5*. Detailed ophthalmic examinations included anterior segment examination by slit lamp biomicroscopy, measurement of corneal thickness using pachymetry, retinoscopy, color photography, fluorescein angiography, and optical coherence tomography (OCT). A systemic workup including full blood count, coagulation screen and renal function analyses was performed.

### Clinical samples

BCS-affected ocular tissue was obtained from the Department of Histopathology, Manchester Royal Infirmary. Human ocular tissue samples from control individuals were obtained from the Manchester Eye Bank (Table 1). Informed written consent and ethics committee approval was granted (14/NW/1495).

**Table 1:** Clinical samples used for study

Clinical samples	Age	Sex	Pathology	*PRDM5* mutation	Functional consequence of mutation	Application	Reference
						IHC – human eye	
						WB – skin fibroblasts	
						IF – skin fibroblasts	
P1	10	M	BCS	Δ 9–14 exons	Shortened, internally deleted protein product	IHC/WB	[4]
P2	21	F	BCS	Δ 9–14 exons	Shortened, internally deleted protein product	IHC/Q-PCR/WB	[4]
P3	8	M	BCS	Arg590*	Truncated protein product	Q-PCR/WB	[4]
P4	26	F	BCS	p.Glu134*	Presumed null	Case report/IF	
Control post-mortem eye #1	70	F	None	Wild-type		IHC	
Control post-mortem eye #2	58	M	None	Wild-type		IHC	
Control skin fibroblasts #1	12	M	None	Wild-type		IF/WB	[4]
Control skin fibroblasts #2	19	F	None	Wild-type		IF	[4]

*IHC* Immunohistochemistry, *WB* Western blotting, *IF* Immunofluorescence

### Genetic analysis

The open reading frames of *ZNF469* and *PRDM5* were sequenced as described [3, 4]. Variants identified in *PRDM5* were checked against control data sets including dbSNP (Build 137) (http://www.ncbi.nlm.nih.gov/SNP), the 1000 Genomes Project (May 2012 release) (http://browser.1000genomes.org/index.html), and the NHLBI Exome Sequencing Project (http://evs.gs.washington.edu/EVS).

### Immunoblotting

Fibroblast cell lysis and preparation of nuclear extracts was performed according to Schnitzler GR [7]. Total protein content was quantified using a BioRad protein quantification BCA assay (BioRad Laboratories). Skin fibroblasts nuclear extracts were subjected to standard SDS-PAGE using a custom-made antibody PRDM5 Ab2 [6, 8] at a concentration of 1 μg/ml, and GAPDH at a concentration of 2 μg/ml (Santa Cruz sc-47724) on equal amounts of nuclear fraction protein. Membranes were blocked with TBST (0.1 % Tween 20) containing 5 % non-fat dry milk, and incubated with primary antibodies overnight. Visualization was performed with an enhanced chemiluminescence western blotting kit (Cell Signalling Technologies #7003).

### Histology and immunohistochemistry

Histological analysis was carried out in accordance with standard diagnostic protocols. 4 μm paraffin-embedded slides were stained with hematoxylin and eosin and elastin with van Gieson. Immunohistochemistry was performed using PRDM5 Ab2 [6, 8] and mouse monoclonal antibodies against collagen I (ab90395, Abcam); collagen III (ab6310, Abcam) and collagen IV (ab6311, Abcam). PRDM5 AB2 epitope is situated within the region corresponding to N- terminal amino acids 60–142. Staining was performed on a Ventana Benchmark XT Automated Immunostaining Module (Ventana Medical Systems) together with the XT *ultra*View Universal Red Alkaline Phosphatase detection system for all antibodies except PRDM5, where DAB was used as the chromogen. Antigen retrieval was performed separately using heat-induced antigen retrieval for PRDM5, and no pre-treatment for collagens I, III and IV. Primary antibodies were diluted in Dako REAL™ Antibody Diluent (Dako, Agilent Technologies, UK) to the indicated optimal dilutions of 3.5 μg/ml for PRDM5; 3 μg/ml for collagen I; 10 μg/ml for collagen III; 2.5 μg/ml for collagen IV. Sections of patient eye tissue were processed in parallel with the control tissue and were collected, sorted and fixed in an identical manner. Tissue section slides were masked for origin and scored for detection of cells showing nuclear PRDM5 staining subjectively by an independent human observer using a binary scale (positive or negative). Tissues were considered positive when >20 % of the cells displayed nuclear PRDM5 staining [8].

### Cell culture and immunofluorescence (IF)

Polyclonal rabbit anti-fibronectin (FN) antibody, mouse anti-tenascin monoclonal antibody (clone BC-24), recognizing all the tenascins (TNs), and TRITC- conjugated rabbit anti-goat antibody were from Sigma Aldrich; mouse anti-α5β1 (clone JBS5) and anti-α2β1 (clone BHA.2) integrin monoclonal antibodies; and goat anti-type I collagen, anti-type III collagen and anti-type V collagen antibodies were from Millipore Chemicon Int. Inc. (Billerica, MA). FITC- and TRITC-conjugated goat anti-rabbit and anti-mouse secondary antibodies were from Calbiochem-Novabiochem Int. (San Diego, CA, USA). Antibody dilutions were: anti-tenascin and anti-α5β1: 2 μg/ml; anti-α2β1: 4 μg/ml; anti-FN and anti-type I collagen, 10 μg/ml; anti-type III and type V collagen: 20 μg/ml. Primary dermal fibroblast cultures were established from skin biopsies by routine procedures, maintained and harvested as described [9, 10]. 1.0 × 10^5^ cells were grown for 48 h on glass coverslips, fixed in methanol and incubated with the specified antibodies as reported [9, 10]. For analysis of integrins, cells were fixed in 3 % paraformaldehyde and 60 mM sucrose, and permeabilized in 0.5 % Triton X-100. Cells were reacted for 1 h at room temperature with 1 μg/ml anti-α5β1 and anti-α2β1 integrin monoclonal antibodies. Cells were subsequently incubated with 10 μg/ml FITC- or TRITC-conjugated secondary antibodies. IF signals were acquired by a CCD black/white TV camera (SensiCam-PCO Computer Optics GmbH, Germany) mounted on a Zeiss fluorescence-Axiovert microscope, and digitalized by Image Pro Plus program (Media Cybernetics, Silver Spring, MD).

### Quantitative PCR

Extracted total RNA was reverse-transcribed into single-stranded cDNA using a High Capacity RNA-to-cDNA Kit (Life Technologies, Paisley, UK), according to the manufacturer’s instructions. RT-PCR and data analysis was performed as previously described [4]. The assay numbers for the mRNA endogenous control (*GAPDH*) and target gene were: *GAPDH* (Hs02758991_g1*) and *ITA8* (Hs00233321_m1*) (Life Technologies). Cycles to threshold (CT) values were determined for each sample and its matched control and relative mRNA expression levels determined by the 2^−ΔΔ*Ct*^ method, providing the fold change value [11]. Error bars representing 95 % confidence intervals around the mean are represented for all experiments. P-values were derived using the 2-tailed *T*-test with significance level set at 0.01 to compare results between mutant and wild-type cells. One-way ANOVA and Dunnett’s multiple comparison posttest using mean values and standard error were also performed on fold change means in all groups assessed.

## Results

### *PRDM5* mutations, functional consequences, and associated phenotypes

**Fig. 1:**
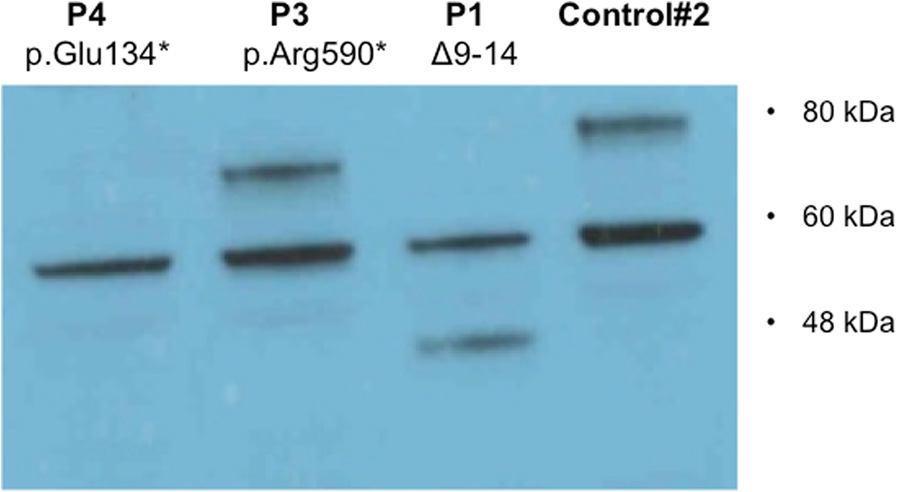
Functional effects of *PRDM5* mutations. Western blot using antibody PRDM5 Ab2 in skin fibroblasts from patients P1 with deletion of exons 9–14 and P3 with p.Arg590* (described by Burkitt-Wright et al. [4]), and an unaffected age-matched control (WT). The in-frame deletion of exons 9–14 (P1) produces a smaller protein of approximately 45 kDa that is consistent with the predicted mass. The p.Arg590* nonsense mutation is in the last exon of *PRDM5* and results in a protein of approximately 70 kDa, consistent with the predicted mass of the PRDM5 protein resulting from the truncated transcript. The WT protein from the unaffected control gives a band of the expected mass (78 kDa). A non-specific band at 60 kDa is present in all samples. P4 protein from a patient with the presumed null mutation p.Glu134* does not produce any bands other than the non-specific band at 60 kDa

**Fig. 2:**
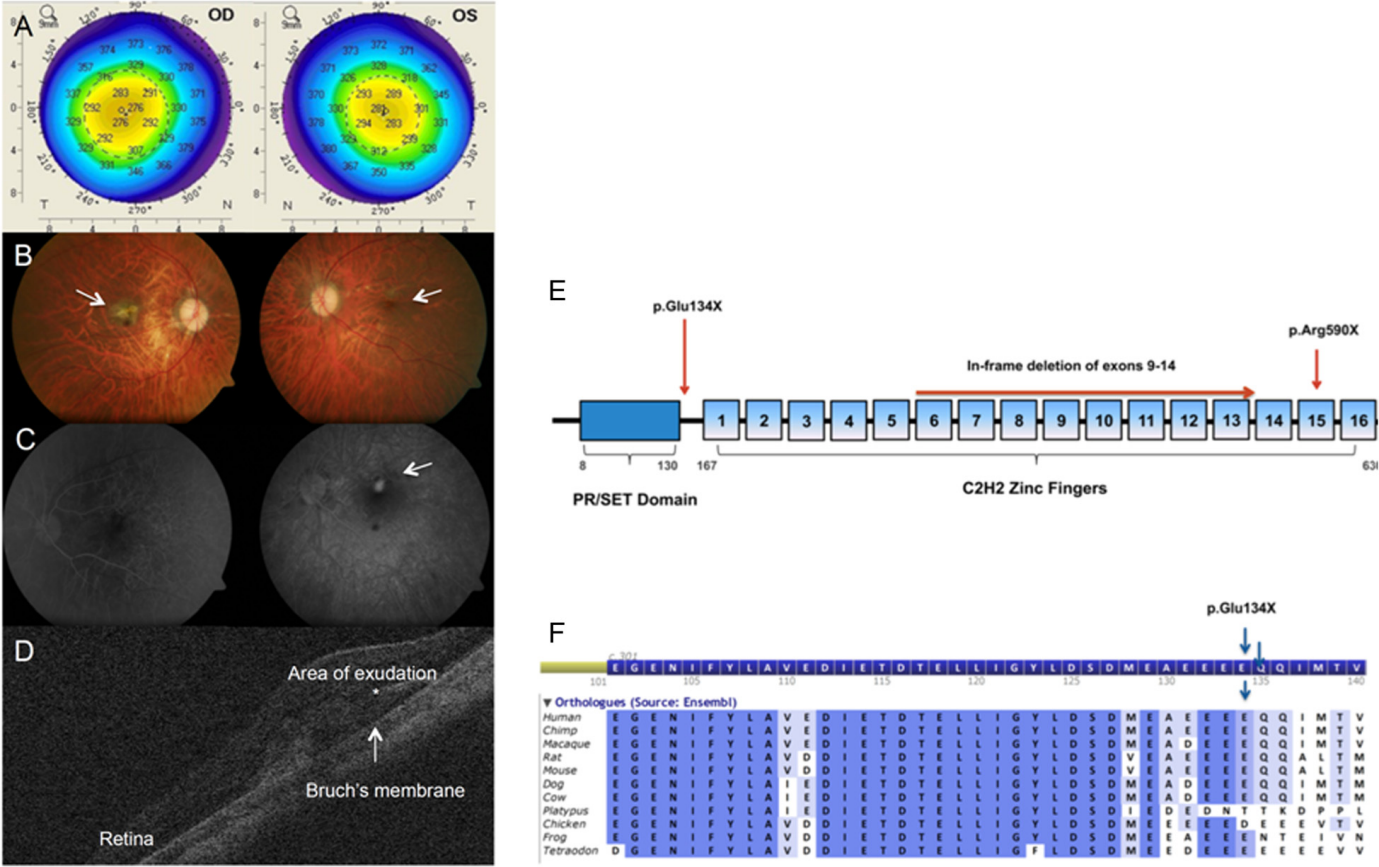
A case of *PRDM5*-associated disease presenting with myopic choroidal neovascularization. **a**. Corneal topography of right eye (OD) and left eye (OS) in patient P4 demonstrating marked corneal thinning, with central corneal thickness measuring 276 μm right eye and 281 μm left eye. **b**. Retinal photograph of RE (*upper left panel*) and LE (*upper right panel*) demonstrating evidence of disciform-like scarring in the macular region of the RE (*arrow*), and active choroidal neovascularization in the LE (*arrow*) leading to retinal exudation. Disc pallor and choroidal thinning are also present, related to the high refractive error of P4. **c**. Two fluorescein angiogram images of the LE taken at the time of presentation. Early phase (*left panel*) and late phase (*right panel*). An area of exudation indicative of vascular leakage into the retina is evident, suggestive of predominantly classic choroidal neovascularization. **d**. Optical coherence tomography scan of the LE at baseline demonstrating the presence of vascular exudation into the retina (*). **e**. Schematic of PRDM5 protein showing the novel mutation p.Glu134*; *PRDM5* deletion exons 9–14; and p.Arg590*. **f**. Amino acid conservation in 11 species upstream and flanking the mutation p.Glu134*. The alignment was generated using Alamut v2.3 (Interactive Biosoftware, 2013)

A summary of clinical samples used in this study is shown in Table 1. The mutation *PRDM5* Δ9–14, carried by P1 and P2 (whose clinical details are described in Burkitt-Wright et al. [4]), is an in-frame deletion mutation that we show here results in the production of a smaller, internally deleted protein confirmed by western blot analysis on skin fibroblasts of P1 (Fig. 1). The mutation p.Arg590* (P3) (whose description is also provided in Burkitt-Wright et al. [4]), also results in a protein truncation confirmed by western blot analysis on skin fibroblasts (Fig. 1). P4 is a 26-year-old woman who presented with decreased vision in her left eye (LE) in the context of normal birth, developmental and family history. Of note she had been seen four years previously in an identical setting with similar symptoms in her right eye (RE), resulting in RE permanent visual loss. On examination best-corrected visual acuity measured 0.16 RE and 0.4 LE (Snellen decimal equivalent). Blue sclera, corneal thinning with central corneal thicknesses of 276 μm RE and 281 μm LE (Fig. 2a), and high myopia were present (spherical equivalent refraction: −12.50 RE and −13.75 LE). Axial lengths were increased in both eyes (28 mm) (IOLMaster, Carl Zeiss Meditec Inc, Jena, Germany). Dilated retinal examination demonstrated a disciform-like scar in her RE and evidence of active choroidal neovascularization (CNV) in her LE (Fig. 2b and c, arrows), leading to retinal exudation (Fig. 2d,*). Management of CNV consisted of three consecutive monthly intravitreal injections of the anti-vascular endothelial growth factor (VEGF) agent ranibizumab (Lucentis, Novartis, Basel, Switzerland), achieving a complete resolution of the exudation and significant VA improvement to 0.70. In a follow-up period of 5 years there has been no disease recurrence. Systemic examination showed marfanoid habitus, scoliosis, arachnodactyly and hyperextensible joints. Genetic analyses revealed a novel homozygous nonsense mutation in *PRDM5*, c.400G > T p.Glu134*, confirming a diagnosis of BCS. The mutated nucleotide is in exon 4 (Fig. 2e) and is predicted to result in nonsense-mediated decay. This variant was not present in dbSNP132, the Exome Variant Server, or the 1000 Genomes Project (all accessed January 5^th^ 2015). Western blot analysis on skin fibroblasts of P4 did not detect a protein product, however overlap between the location of the mutation, and location of the antibody epitope was present.

### PRDM5 is expressed in the adult human cornea and retina

**Fig. 3:**
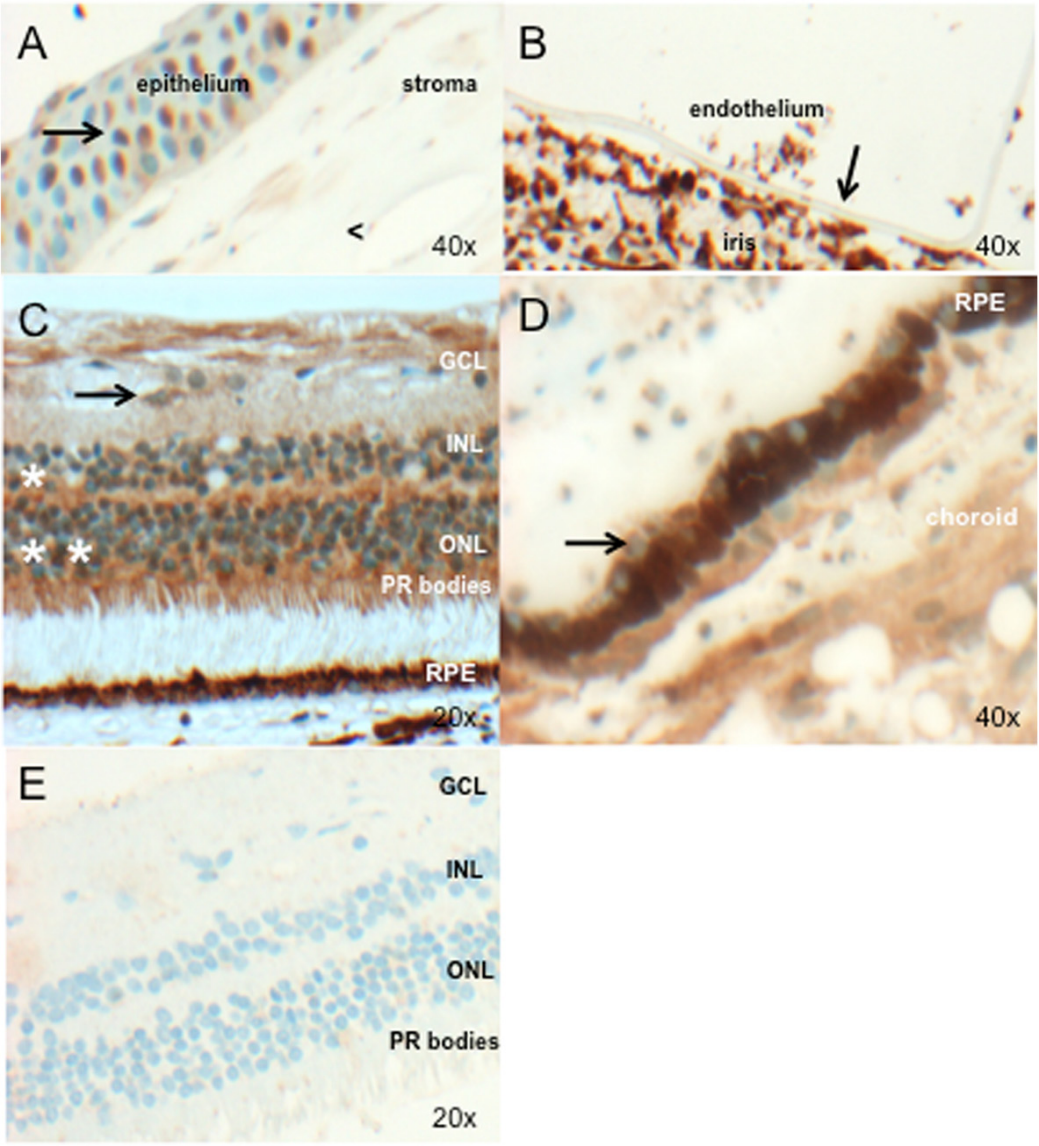
PRDM5 expression in the adult human eye (**a**–**d**) (control #2, Table 1). Image objective magnifications (OM) are shown. PRDM5 staining is brown (obtained using DAB as the chromogen). **a**. Nuclei of the corneal epithelium are positive for PRDM5 (*arrow*). The corneal stroma shows mild cytoplasmic staining, but nuclei are negative (<). **b**. PRDM5 is not expressed in the corneal endothelium (*arrow*) (in this image the endothelium has become detached from the overlying stroma). **c**. Positive PRDM5 nuclear and cytoplasmic staining in the inner (*) and outer nuclear layers (**), with cones staining more strongly than rods. Ganglion cell cytoplasm are also positive (*arrow*). Ocular tissue from patient P1 with deletion exons 9–14 is shown in (**e**). GCL, ganglion cell layer; INL, inner nuclear layer; ONL, outer nuclear layer; PR, photoreceptors, RPE; retinal pigment epithelium. **d**. Retinal pigment epithelium cell nuclei (*arrow*) appear negative. **e**. PRDM5 staining in P1 with *PRDM5* deletion exons 9–14, demonstrating loss of PRDM5 nuclear and most cytoplasmic staining in the retina associated with loss of antibody epitope

Investigation of PRDM5 expression by immunohistochemistry shows nuclear PRDM5 expression in the corneal epithelium (Fig. 3a) and extensive nuclear and cytoplasmic PRDM5 expression in the retina (Fig. 3c), with absent expression in the retinal pigment epithelium (Fig. 3e, arrow) (control post-mortem eye #2). PRDM5 staining was also not apparent in Bruch’s membrane. Expression was seen in cells of neuroectodermal origin particularly the retina, with the exception of nuclei of the smooth muscle of the ciliary body (mesodermal origin) and nuclei of the corneal epithelium (surface ectodermal origin) (Fig. 3 and Additional file 1: Table S1). Immunohistochemistry using PRDM5 Ab2 in P1 demonstrates loss of PRDM5 retinal nuclear and cytoplasmic staining (Fig. 3e).

### Structural abnormalities in Bruch’s membrane in BCS patients

**Fig. 4:**
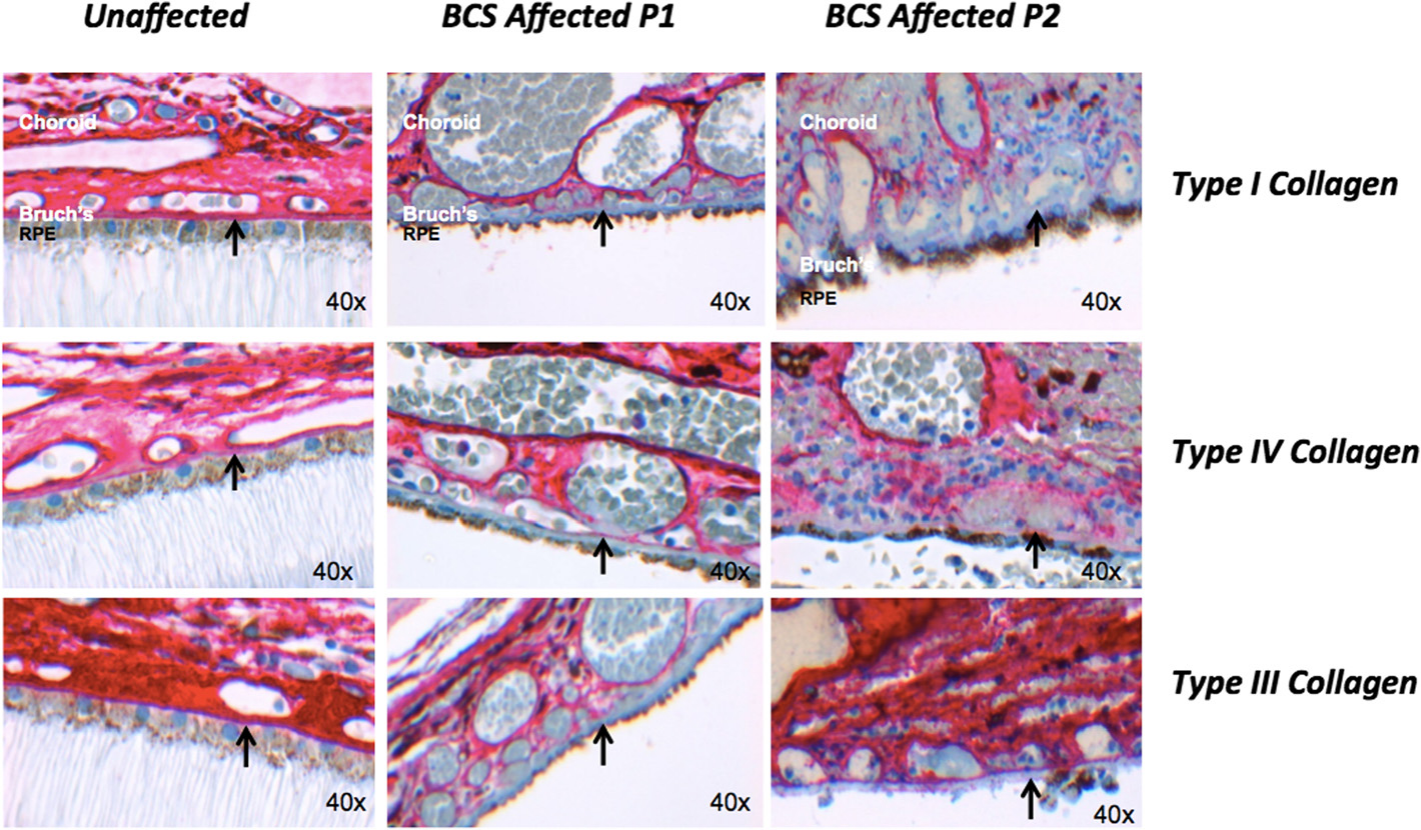
Changes in extracellular matrix collagens in Bruch’s membrane in PRDM5-associated disease. Immunohistochemistry performed for collagens I, III and IV (red stain, obtained using the XT *ultra*View Universal Red Alkaline Phosphatase detection system) in retinas of a control individual, and BCS patients P1 and P2 (OM 40×). Absence of staining for collagens I, III and IV in Bruch’s membrane (*arrow*) is present in sample P1, and absent staining for collagen I with reduced staining for collagens III and IV in P2. Images were recorded and processed identically to allow direct comparisons to be made between them

Two patients, cousins, aged 10 (P1) and 21 (P2) with *PRDM5*-associated disease (previously described by Burkitt-Wright et al. [4]) were studied in order to assess whether there are any functional consequences to their retinas. As collagen expression is associated with PRDM5 levels, we studied the expression of PRDM5, collagens I, III, IV and elastin in Bruch’s membranes of eyes from these two subjects. Both patients carried a large homozygous deletion (Δ exons 9–14) in *PRDM5.* The axial lengths of the two eyes were 20 mm (P1) and 21.8 mm (P2), determined histologically. We found absent or decreased staining in Bruch’s membrane for collagens type I, III and IV in both BCS samples versus a control sample (Fig. 4). Van Gieson staining demonstrated normal elastin staining of Bruch’s membrane in both samples (Additional file 1: Figure S1). No histological evidence of breaks in Bruch’s membrane was evident.

### Extracellular matrix abnormalities in skin fibroblasts from BCS patient P4 with the novel *PRDM5* mutation p.Glu134*

**Fig. 5:**
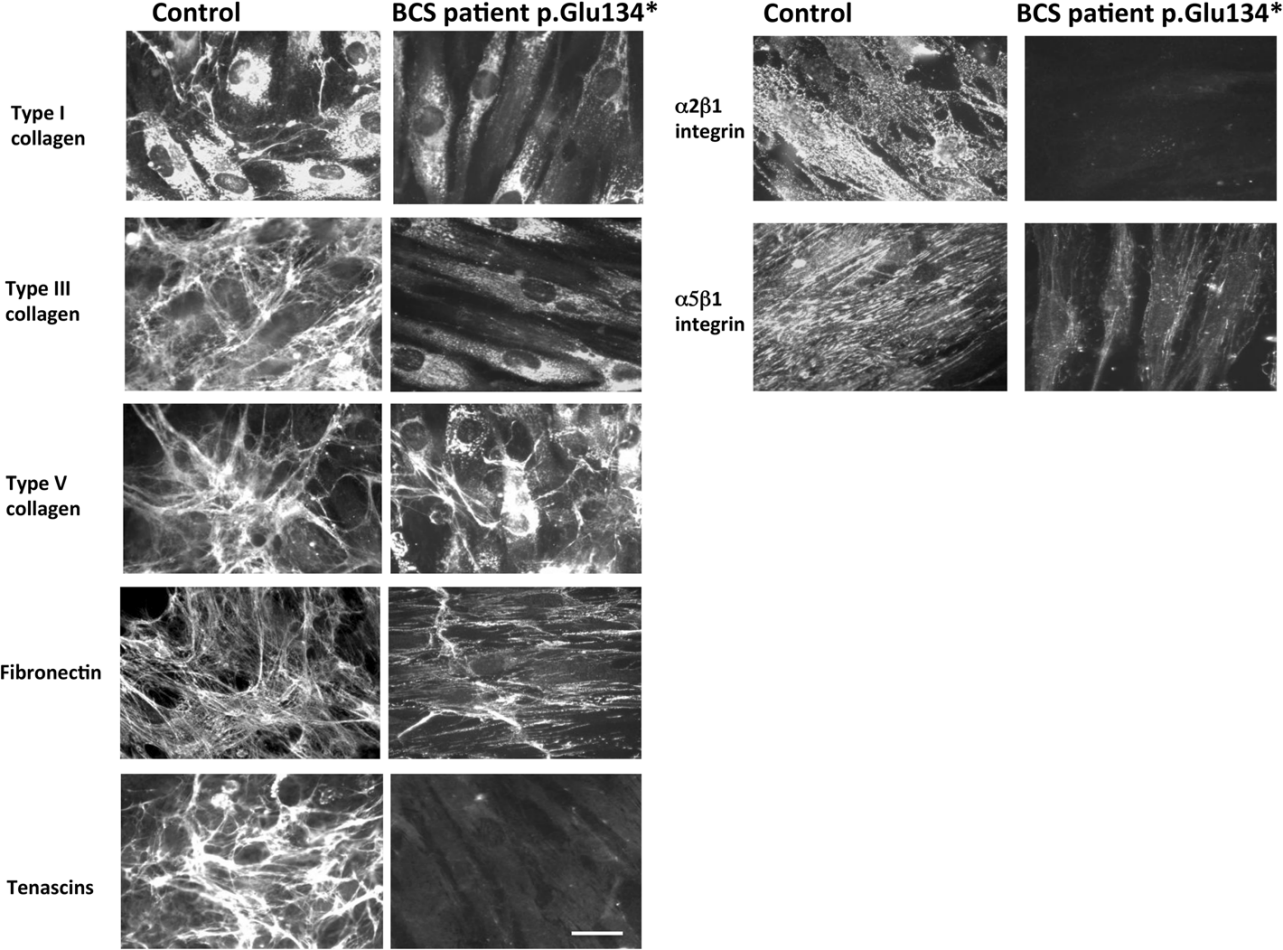
Expression of ECM components in dermal fibroblasts expressing or lacking PRDM5. Indirect immunofluorescence microscopy performed on dermal fibroblasts from a control (#2) and BCS patient P4 carrying the *PRDM5* p.Glu134* mutation for collagens I, III and V, fibronectin, tenascins and integrin receptors α2β1 and α5β1. In control cells, collagen I is primarily expressed in the cytoplasm, with only limited expression extracellularly. Collagen I labelling is substantially reduced in *PRDM5* mutant cells. Collagen III appears well organized in the ECM of control cells but was absent in the mutant fibroblasts where only diffuse cytoplasmic staining was visible. Furthermore, disarray of collagen V was evident with cytoplasmic accumulation and reduced extracellular matrix in the *PRDM5* mutant cells. Expression of the collagen integrin receptor α2β1 was essentially abolished, with marked reduction in the fibronectin integrin receptor α5β1 and disorganization of fibronectin matrix, in PRDM5 mutant cells compared to control cells. Tenascins were organized in an abundant extracellular matrix in control cells whereas they were not detectable in the PRDM5 mutant cells. 1 cm on the image scale corresponds to 16 μm. Images were all recorded under identical parameters to allow for direct comparison

Indirect immunofluorescence performed on skin fibroblasts from patient P4 showed downregulation of structural collagens I and III, fibronectin, all the tenascins (detected by a single antibody), and integrin receptors α2β1 and α5β1. The expression of type V collagen was disorganized in comparison to the control (Fig. 5).

## Discussion

*PRDM5*-related disease is known to affect the cornea, skin and joints [1–5]. Here we show that PRDM5 localizes not only in the human cornea, but is also widely expressed in the retina. PRDM5 expression has been reported to be predominantly nuclear, for example in intestinal crypts where stem cells reside, with cytoplasmic expression in some tissues, including colonic villi [8]. We show both cytoplasmic and nuclear PRDM5 expression in the retina. We show reduced expression of major collagenous components of Bruch’s membrane [12] (Additional file 1: Figure S2) in two patients with a deletion of exons 9–14 of *PRDM5* (Fig. 4). An association between PRDM5 and altered collagen expression has been shown in previous studies [4–6]. Here we show that PRDM5 mutations lead to notable differences in the expression of ECM proteins in Bruch’s membrane that may impinge on its structural integrity.

We identified myopic choroidal neovascularization (CNV) [13] in a 26-year old lady with PRDM5-related BCS, suspected to have the disease due to the presence of corneal thinning and marfanoid features. High myopia is a significant risk factor for the development of both CNV and retinal detachment [13] and has been described in a number of patients with BCS [2]. Patients with BCS may therefore benefit from daily monocular monitoring with an Amsler chart, to detect early metamorphopsia (visual distortion), with urgent referral to an ophthalmologist if metamorphopsia develops. CNV has been described in a number of connective tissue disorders including pseudoxanthoma elasticum [14], Beals-Hecht syndrome [15], Ehlers-Danlos syndrome [16], and osteogenesis imperfecta [17]. Associations between connective tissue disease and CNV have however seldom been investigated at the histological and cellular levels. *PRDM5* mutations may further contribute to weaknesses at the level of Bruch’s membrane caused by myopia, a hypothesis consistent with our immunohistochemical results, although IHC was only performed on two patients with a *PRDM5* Δ9-14, which results in the production of a truncated protein product. Retinal basement membrane abnormalities have been also noted upon ultrastructural studies of retinas from patients with Alport syndrome, caused by mutations in different transcripts of the collagen IV gene [18].

We looked at expression of fibronectin, integrins and tenascins in dermal fibroblasts. Fibronectin is present in Bruch’s membrane (Additional file 1: Figure S2), and integrins α2β1 and α5β1 are the major integrin receptors for collagen and fibronectin, respectively. Integrin α8 is the major tenascin receptor [19] and homozygous mutations in tenascin X cause a subtype of Ehlers-Danlos syndrome [20, 21]. When present in dermal fibroblasts, the *PRDM5* p.Glu134* mutation results in the absence of tenascin staining (Fig. 5). These findings are consistent with our previous study examining patient fibroblasts from patients with the Δ9–14 and p.Arg590* *PRDM5* mutations [4]. Here we confirm that expression of integrins α2β1, α5β1 is markedly reduced (Fig. 5). We also note reduced RNA expression levels of integrin α8 in two patients (Additional file 1: Figure S3). Integrin α8 is the major tenascin receptor [19]. Tenascins are a family of four extracellular matrix proteins, tenascin X and C are major isoforms expressed in ocular tissues [22]. The altered expression of collagen V in fibroblasts lacking PRDM5, together with the absence of tenascins, is reminiscent of a subtype of autosomal recessive Ehlers-Danlos syndrome characterized by tenascin X deficiency [20, 21]. Tenascin X is a large ECM glycoprotein abundantly expressed during development and in adult tissue strongly associated with ocular basement membranes (Bowman’s layer and Descemet’s membrane in particular) [22]. Our data suggests that PRDM5 may play a role in the regulation of collagen, integrin and tenascin expression, proteins that participate in ocular basement membrane development including Bruch’s membrane [12, 22]. A role for PRDM5 as a direct activator of collagen genes has been reported with direct binding of PRDM5 in conjunction with RNA polymerase II shown in a ChIP-sequencing experiment performed on murine MC3T3 cells (6). This role is also supported by the observation of a significant downregulation of structural collagens in fibroblasts of patients with BCS2 (4). While it is also possible that the basement membrane structural abnormalities observed in Bruch’s membrane also involve the corneal endothelial and/or epithelial basement membranes, the presence of extreme corneal scarring and ECM changes linked to tissue remodelling precluded this analysis.

## Conclusions

PDRM5-related disease is known to affect the cornea, skin and joints. Our study shows expression of PRDM5 in the human cornea and retina, and demonstrates downregulation of major structural components of Bruch’s membrane in the eyes of two patients with BCS type 2. These findings suggest that ECM abnormalities in *PRDM5*-associated disease are more widespread than previously reported.

## Additional file

Additional file 1: Table S1. Ocular PRDM5 expression and embryonic origin of ocular structure. **Figure S1.** Elastin staining in Bruch’s membrane in PRDM5-associated disease. **Figure S2.** The 5 layers of Bruch’s membrane (modified from Booji et al, 2010) [15]. **Figure S3.** qPCR assessment of target gene *ITGA8* demonstrating fold change in mRNA expression in dermal fibroblasts isolated from BCS patients with different mutations: *PRDM5* p.Arg590* (P3) and *PRDM5* internal deletion of exons 9 to 14 (P1), versus sex and age-matched control fibroblast cell lines in the logarithmic scale (Log2RQ)*.* (DOCX 5201 kb)
