# Supplementary material for: Bruch’s membrane abnormalities in PRDM5-related brittle cornea syndrome
Source: Orphanet J Rare Dis. 2015 Nov 11;10:145. doi: 10.1186/s13023-015-0360-4 (PMC4642625; doi:10.1186/s13023-015-0360-4)
Supplement: Additional file 1: Table S1. — Ocular PRDM5 expression and embryonic origin of ocular structure. Figure S1. Elastin staining in Bruch’s membrane in PRDM5-associated disease. Figure S2. The 5 layers of Bruch’s membrane (modified from Booji et al, 2010) [15]. Figure S3. qPCR assessment of target gene ITGA8 demonstrating fold change in mRNA expression in dermal fibroblasts isolated from BCS patients with different mutations: PRDM5 p.Arg590* (P3) and PRDM5 internal deletion of exons 9 to 14 (P1), versus sex and age-matched control fibroblast cell lines in the logarithmic scale (Log2RQ). (DOCX 5201 kb) [file 13023_2015_360_MOESM1_ESM.docx]

**Additional Files:**

**Supplementary Table1**. **Ocular PRDM5 expression and embryonic origin of ocular structure.**

PRDM5 is expressed predominantly in tissues of neuroectodermal origin.

| **Superficial epithelium** | | **Mesenchyme** | | | | **Neuro-epithelium** | |
| --- | --- | --- | --- | --- | --- | --- | --- |
| Ectoderm | PRDM5 | Neural crest | PRDM5 | Mesoderm | PRDM5 | Neural plate | PRDM5 |
| Corneal epithelium | √ | Corneal stroma | **-** | Blood vessel endothelium | **-** | Sensory retina | √ |
|  |  |  |  |  |  | INL nuclei | √ |
|  |  | Corneal endothelium | **-** |  |  | ONL nuclei | √ |
|  |  |  |  |  |  | GCL nuclei | √ |
| Lens | - |  |  | Bruch’s membrane | **-** | Nerve fiber layer | √ |
|  |  |  |  |  |  | Rods and cones | √ |
|  |  |  |  |  |  | Pigmentary retina | - |
|  |  |  |  |  |  | RPE nuclei | - |
|  |  |  |  |  |  | RPE cytoplasm | - |
| Bruch’s membrane | - | Iris stroma | **-** |  |  | Lens capsule | √ |
|  |  | Dura mater | **-** |  |  |  |  |
|  |  | Smooth muscle ciliary body | √ |  |  |  |  |

**Supplementary Figure 1. Elastin staining in Bruch’s membrane in PRDM5-associated disease.**

Elastin expression detected by Van Gieson staining, shown in purple (Objective magnifications (OM)) are shown). **A.** Retina from an unaffected individual (#2) **B.** Choroid and RPE from patient P1 with Δ exons 9-14 *PRDM5*. **C.** Choroid and RPE from patient P2 with Δ exons 9-14 *PRDM5*. The neural retina in C and D were detached. Elastin staining in Bruch’s membrane (arrow) is comparable between the control (C) and the two BCS patients (P1 and P2).

**Supplementary Figure 2: The 5 layers of Bruch’s membrane (modified from Booji *et al, 2010)* [15].** Major structural proteins are represented within each layer. Proteins demonstrating significant down-regulation in BCS-type 2 retinas or skin fibroblasts are highlighted with an arrow. The basement membrane of the retinal pigment epithelium is composed of collagens type IV and V, laminin, and heparan and chondroitin/dermatan sulphate (HSPG). Of these proteins, we studied the expression of collagen type IV and demonstrated large downregulation of collagen type IV in PRDM5-associated disease. The inner collagenous layer of Bruch’s membrane is composed primarily of collagens type I, III, V and fibronectin, all of which were either downregulated in Bruch’s membrane of patient retinas carrying the deletion of exons 9-14 *PRDM5* mutation (P1 and P2), or the skin fibroblasts of a patient lacking PRDM5 (P4) (collagens type I and III). The lack of PRDM5 also appeared to result in the disorganized expression of collagen type V in skin fibroblasts. The elastin layer consists predominantly of elastin fibres, collagens type IV and VI, collagen type XVIII and fibronectin. The basement membrane of the choriocapillaris is predominantly composed of laminin, heparan sulphate and collagens type IV, V and VI. BM: Bruch’s membrane; RPE: retinal pigment epithelium.


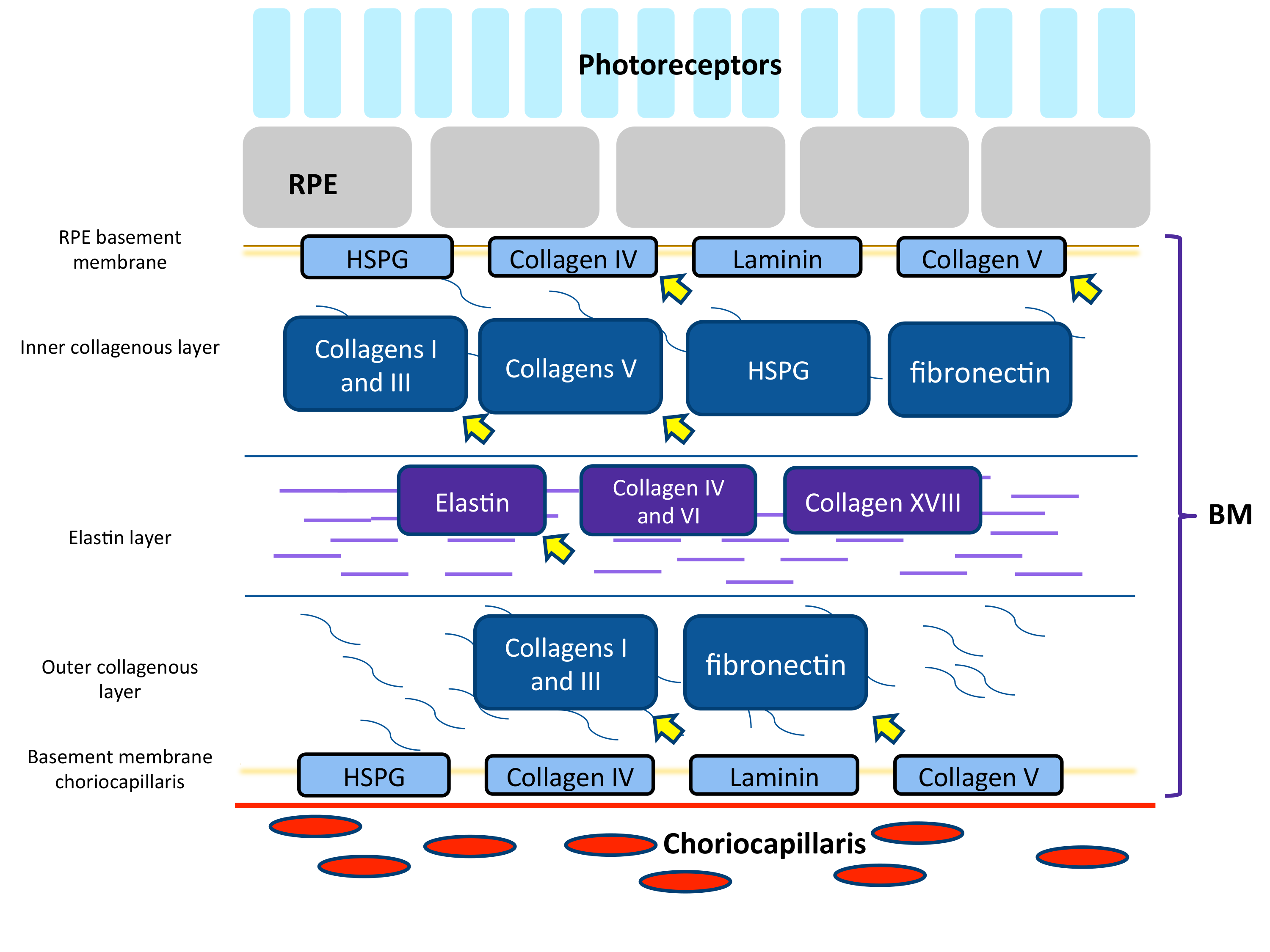


**Supplementary Figure 3**. qPCR assessment of target gene *ITGA8* demonstrating fold change in mRNA expression in dermal fibroblasts isolated from BCS patients with different mutations: *PRDM5* p.Arg590* (P3) and *PRDM5* internal deletion of exons 9 to 14 (P1), versus sex and age-matched control fibroblast cell lines in the logarithmic scale (Log2RQ)*.* mRNA levels were normalized to *GAPDH* expression as described in the methods section. The Y-axis represents fold change (RQ) in gene expression determined by the 2^−ΔΔ^*^Ct^* method. The X-axis shows the target gene assessed. Error bars represent the 95% confidence interval around the mean. *ITGA8* transcript levels in cell lines of patients with *PRDM5* mutations are decreased relative to control fibroblast cell lines. The p-value for the paired two-tailed t-test reaches statistical significance (P<0.01).
